# Supplementary material for: Meta-analysis on the effects of moderate-intensity exercise intervention on executive functioning in children
Source: PLoS One. 2023 Feb 22;18(2):e0279846. doi: 10.1371/journal.pone.0279846 (PMC9946206; doi:10.1371/journal.pone.0279846)
Supplement: S1 File — (DOC) [file pone.0279846.s001.doc]

In this study, PubMed and China National Knowledge Infrastructure were used as examples, and the complete search strategy is shown in Tables 1-2.

**Table 1 PubMed search strategy.**

| **Number** | **Search Content** |
| --- | --- |
| #1 | "exercise"[MeSH Terms] |
| #2 | "exercises"[Title/Abstract] |
| #3 | "physical activity"[Title/Abstract] |
| #4 | "physical activities"[Title/Abstract] |
| #5 | "physical exercise"[Title/Abstract] |
| #6 | "physical exercises"[Title/Abstract] |
| #7 | "acute exercise"[Title/Abstract] |
| #8 | "acute exercises"[Title/Abstract] |
| #9 | "aerobic exercise"[Title/Abstract] |
| #10 | "aerobic exercises"[Title/Abstract] |
| #11 | #1 OR #2 OR #3 OR #4 OR #5 OR #6 OR #7 OR #8 OR #9 OR #10 |
| #12 | "child"[Title/Abstract] |
| #13 | "children"[Title/Abstract] |
| #14 | #12 OR #13 |
| #15 | "executive Function"[Mesh] |
| #16 | "inhibitory control"[Title/Abstract] |
| #17 | "working memory"[Title/Abstract] |
| #18 | "cognitive flexibility"[Title/Abstract] |
| #19 | #15 OR #16 OR #17 #18 |
| #20 | "randomized controlled clinical trial"[Publication Type] |
| #21 | " trial"[Publication Type] |
| #22 | #20 OR #21 |
| #23 | #11 AND #14 AND #19 AND #22 |

**Table 2. China National Knowledge Infrastructu research strategy**

| **Number** | **Search Content** |
| --- | --- |
| #1 | subject terms='运动' OR free words='运动+锻炼+体力活动+体育锻炼+急性运动+有氧运动' |
| #2 | subject terms='学生' OR free words= '儿童 + 学生 + 小学生 + 学龄儿童' |
| #3 | subject terms='执行功能' OR free words='执行功能 + 抑制控制 + 工作记忆 + 认知灵活性' |
| #4 | subject terms='实验研究' OR free words='实验研究 + 随机对照 + 临床试验' |
| #5 | #1 AND #2 AND #3 AND #4 |
