# Supplementary material for: Meta-analysis on the effects of moderate-intensity exercise intervention on executive functioning in children
Source: PLoS One. 2023 Feb 22;18(2):e0279846. doi: 10.1371/journal.pone.0279846 (PMC9946206; doi:10.1371/journal.pone.0279846)
Supplement: S2 Checklist — (DOCX) [file pone.0279846.s003.docx]

**Identification of studies via databases and registers**

Records identified from:

Chinese National Knowledge Infrastructure(n=102);

Wanfang database(n=287);

China Science and Technology Journal Database(n=9);

Web of science(n=305);

Pubmed(n=91);

Databases (n=794 )

Records removed *before screening*:

Duplicate records removed (n=534)

Records removed for other reasons (n =42 )

**Identification**

Records excluded overview (n=37)

Literature in review category (n=46)

Records screened (n=218 )

Literature with research objects which do not meet the requirements (n=47)

Excluded by abstracts (n=135)

**Screening**

Literature with experiments that are not randomized and controlled (n=25)

Literature with the intervention measures which do not meet the requirements (n=16)

Excluded by study objects

(n=88)

Literature with incomplete outcome indexes (n=19)

Excluding Jadad score ≤ 2 (n=6)

Excluded by study process

(n=47)

**Included**

Reports of included studies

(=22 )

*Consider, if feasible to do so, reporting the number of records identified from each database or register searched (rather than the total number across all databases/registers).

**If automation tools were used, indicate how many records were excluded by a human and how many were excluded by automation tools.

*From:*  Page MJ, McKenzie JE, Bossuyt PM, Boutron I, Hoffmann TC, Mulrow CD, et al. The PRISMA 2020 statement: an updated guideline for reporting systematic reviews. BMJ 2021;372:n71. doi: 10.1136/bmj.n71

For more information, visit: <http://www.prisma-statement.org/>
