# Supplementary material for: Meta-analysis on the effects of moderate-intensity exercise intervention on executive functioning in children
Source: PLoS One. 2023 Feb 22;18(2):e0279846. doi: 10.1371/journal.pone.0279846 (PMC9946206; doi:10.1371/journal.pone.0279846)
Supplement: Attachment — Submitted filename: Response to Reviewers.doc [file pone.0279846.s004.doc]

# Response to Reviewer 1 Comments:

Greetings! Thank you for your valuable comments on the article, the article is revised to clarify the main issues. In what follows, we would like to answer the questions you mentioned and give detailed account of the changes made to the original manuscript.

1.A Pub Med search.

Response 1: The Pub Med database has been retrieved for this research with annotation of the Pub Med database in the paper.

2.Use of PRISMA guidelines.

Response 2: The use of the PRISMA guidelines is described in the following paragraph under " Literature search" and is marked in red on page 5, lines 79-86.

3.A greater emphasis on the strengths of limitations of meta-analysis.

Response 3: We have revised the section "Limitations and Outlook" in the manuscript and highlighted with red font.

4.A greater emphasis on bias and confounding without undue emphasis on the P values.

Response 4: Thank you for pointing out this problem in manuscript. In the revised version, we have modified the discussion section in the manuscript and highlighted with red font.

We appreciate for the reviewer work earnestly on our manuscript, and hope that the correction will meet with approval.

Yours sincerely,

Haixia Wang

16 July 2022

# Response to Reviewer 2 Comments:

Greetings! Thank you for your valuable comments on the article, the article is revised to clarify the main issues. In what follows, we would like to answer the questions you mentioned and give detailed account of the changes made to the original manuscript.

1. Section Literature quality evaluation：“(9 points 5 points, 7 points to get 4 points, 5 articles 3 points)” Please clarify your meaning.

Response 1: We have revised the section "Literature quality evaluation" in the manuscript and highlighted with red font.

2.Section Data analysis:“As an index to determine whether there was any heterogeneity between the studies, the range of I^2^ was from 0% to 100%, and the bigger I^2^ was, the greater the heterogeneity was. I^2^<50% indicated a low heterogeneity between studies, a fixed effect model was adopted to merge the effect size; I^2^≥50% indicated a high heterogeneity between studies, a random effect model was adopted to merge the effect size, and sub-group analysis was performed” Please add references.

Response 2: In our revisions, we have increased the citation of Gong (2021) on page 8, line 144, with red font.

3.Section Basic characteristics of included studies: “A total of 21 literature were included in this study, which were all published in core journals, covering 30 studies. Among them, there were 3 literature (Chen et al.^[11]^, Tomporowski et al. ^[24]^, Yin et al.^[25]^, Yan et al.^[33]^) that included 2 studies, 1 literature (Yan et al.^[14]^) that included 3 studies, and 1 literature (Yin et al.^[19]^) that included 4 studies.A total of 2,466 subjects (1,265 in experimental group and 1,201 in control group), aged 6~12 years old, were included.”

1)How to define “core journals”?

Response (1): We have redescribed the journal sources in the "Basic characteristics of included studies" portion of the manuscript and highlighted it in red on page 9, line 150.

2）The language expression is ambiguous, please clarify the relationship “A total of 21 literature” and “covering 30 studies”.

Response (2): We have redescribed the journal sources in the "Basic characteristics of included studies" portion of the manuscript and highlighted it in red on page 9, line 150-153.

3）“Among them, there were 3 literature (Chen et al.^[11]^, Tomporowski et al. ^[24]^, Yin et al.^[25]^, Yan et al.^[33]^) that included 2 studies,” The author proposed “3 references”, and the results were followed by 4 references, Please check.

Response (3): Thank you for pointing out this problem in manuscript. We have revised the manuscript on page 9, line 151, from 3 references to four references and marked it in red.

4）The author proposed ‘A total of 2,466 subjects’, but inconsistent with the total sample size in Table 1, Please check.

Response (4): We have modified Table 1 and marked it with red font on page 9, line 153.

4. Section Effect of exercise intervention on executive function: “indicating a statistically significant increase in refresh function in children after exercise intervention compared to the control group” and “indicating a statistically significant increase in conversion function in children after exercise intervention”.“after exercise intervention” should be revised to “experimental group”

Response 4: Thank you for your valuable comment. We have revised the term "after exercise intervention" to "experimental group" in the manuscript.

5.Section Basic characteristics of included studies.The amount of physical activity in the control group should be clearly described.

Response 5: We gratefully appreciate for your valuable suggestion. We have appended the physical activity of the control group to Table 1 in the manuscript and marked it with red font.
6. Please check the number of references in Table 1 and Table 4.

Response 6: We have revised the number of references in Table 1 and Table 4. and marked them in red.

7. The World Health Organization has proposed the recommended amount of physical activity for children, which requires that the health benefits of long-term exercise should be better. However, the conclusion of this study recommends that 8-12 weeks of moderate intensity exercise has a good effect on the improvement of executive function, which is in contradiction with the health benefits of long-term exercise. Please draw this conclusion carefully. Please pay attention to this contradiction in the discussion and explain it.

Response 7: This study does not deny the health benefits of long-term exercise; however, the conclusions reached in this research are based on the experimental results of the relevant and included literature that indicate only that 8-12 weeks of moderate intensity exercise is highly effective in improving executive function as explained in the relevant discussion section of the manuscript.

We appreciate for the reviewer work earnestly on our manuscript, and hope that the correction will meet with approval.

Once again, Thank you very much for your comments and suggestions.

Yours sincerely,

Haixia Wang

16 July 2022
