# Supplementary material for: Meta-analysis on the effects of moderate-intensity exercise intervention on executive functioning in children
Source: PLoS One. 2023 Feb 22;18(2):e0279846. doi: 10.1371/journal.pone.0279846 (PMC9946206; doi:10.1371/journal.pone.0279846)
Supplement: Attachment — Submitted filename: Attachments.docx [file pone.0279846.s005.docx]

Line101-102: The three dimensions of the executive function mentioned here, "intrusion function, refresh function and conversion function," are combined with the literature to check whether the language expressions of the three dimensions are accurate and ensure that the translation is correct.

Line389-390: Five databases are mentioned here, and only three databases are listed later. Please improve.

Line 395-396: Please add those findings that support conclusions B and c.
