# Supplementary material for: Meta-analysis on the effects of moderate-intensity exercise intervention on executive functioning in children
Source: PLoS One. 2023 Feb 22;18(2):e0279846. doi: 10.1371/journal.pone.0279846 (PMC9946206; doi:10.1371/journal.pone.0279846)
Supplement: Attachment — Submitted filename: Response to Reviewers.doc [file pone.0279846.s006.doc]

**Dear reviewers:**

Thank you for letter and reviewers' comments on our manuscript entitled "Meta-analysis on the Effects of Moderate-intensity Exercise Intervention on Executive Functioning in Children". Those comments are very helpfulfor revising and improving our paper, as well as the improment guiding significance to our research. We have studied the comments carefully and made corrections which we hope meet with approval. The main corrections are in th manuscript and the responds to the reviewers' comments are as follows.

**Respond to the main points:**

1. your English search terms included "Primary student" which does not have any particular meaning in English. Or "Medium intensity" which is wrong in English and it has to be "Moderate intensity". These are very important to be clarified. Subsequently you mention that "The search strategy is a combination form of separate and mutual, supplemented by literature tracking." What does this mean? You might want to include your search query for clarification (for both Chinese and English searches); it could be as a supplementary document if it is long.

**Response 1：**(a) We re-ran the search after changing the English search terms to exercise, exercises, physical, activity, physical, activities, physical, exercise, physical, exercises, acute exercise, acute exercises, aerobic exercise, aerobic exercises, child, children, executive function, inhibitory control, working memory, cognitive flexibility, randomized controlled clinical trial, trial. On page 5, lines 83-86, the changes are highlighted in red. (b) On page 5, lines 86-88, We changed "the search strategy is a combination form of separate and mutual, supplemented by literature tracking" to "the search strategy was conducted using a combination of subject terms and free words, and references to key documents were manually searched". (c) We have included the complete search strategy in Appendix 1, with examples from the Chinese National Knowledge Infrastructure and PubMed.

2. In the first glance I did not understand what you mean by "refresh" or "conversion" function. I noticed you cited two articles [1-2]. I looked at the review article to see if those words were used. I couldn't find these words. You need to use conventional terms that researchers are familiar with. You need to be clearer in terms of executive functions you looked at such as working memory or inhibition.

**Response 2**: The core vocabulary of inhibition function, refresh function, and conversion function used in the text has been revised to the proper terms inhibitory control, working memory, and cognitive flexibility familiar to researchers in the field. We have specifically asked native English-speaking experts to embellish the text.

3. I still believe you have not responded satisfactorily to the comments of the reviewers, in particular reviewer 1. For example, I do not see much discussion on the "location" in the document.

**Response 3**: In response to reviewer 1's comments in the first revision, we made the following changes.

(1). A PubMed search.

**Response (1)**: We added the PubMed database search to the search of Web of Science, Chinese national knowledge infrastructure, Wanfang database, and Chinese science and technology journal database in the revised version. The changes are highlighted in red on page 5, lines 79-81. A total of 794 articles were retrieved after modifying the search strategy. The PubMed database yielded 91 articles, and the complete search strategy is as follows(Table 1). In Appendix 1, search tactics for other databases are listed.

Table 1 PubMed search strategy.

| **Number** | **Search Content** |
| --- | --- |
| #1 | "exercise"[MeSH Terms] |
| #2 | "exercises"[Title/Abstract] |
| #3 | "physical activity"[Title/Abstract] |
| #4 | "physical activities"[Title/Abstract] |
| #5 | "physical exercise"[Title/Abstract] |
| #6 | "physical exercises"[Title/Abstract] |
| #7 | "acute exercise"[Title/Abstract] |
| #8 | "acute exercises"[Title/Abstract] |
| #9 | "aerobic exercise"[Title/Abstract] |
| #10 | "aerobic exercises"[Title/Abstract] |
| #11 | #1 OR #2 OR #3 OR #4 OR #5 OR #6 OR #7 OR #8 OR #9 OR #10 |
| #12 | "child"[Title/Abstract] |
| #13 | "children"[Title/Abstract] |
| #14 | #12 OR #13 |
| #15 | "executive Function"[Mesh] |
| #16 | "inhibitory control"[Title/Abstract] |
| #17 | "working memory"[Title/Abstract] |
| #18 | "cognitive flexibility"[Title/Abstract] |
| #19 | #15 OR #16 OR #17 #18 |
| #20 | "randomized controlled clinical trial"[Publication Type] |
| #21 | " trial"[Publication Type] |
| #22 | #20 OR #21 |
| #23 | #11 AND #14 AND #19 AND #22 |

(2). Use of PRISMA guidelines.

**Response (2)**: The revised version has been reported under the Preferred Reporting Items for Systematic Reviews and Meta-Analyses (PRISMA) guidelines. It strictly follows the PICOS paradigm for describing the literature and developing criteria for literature search, inclusion, screening and exclusion. The revised version adds a literature quality bias test and a publication bias analysis on pages 16-17.

(3). A greater emphasis on the strengths of limitations of meta-analysis.

**Response (3)**: We revised the "Limitations and Outlook" section of the text by shortening the description of the limitations and highlighting them in red on page 29, lines 431-435.

(4). A greater emphasis on bias and confounding without undue emphasis on the P values.

**Response (4**): Thank you for bringing this to our attention in the manuscript. (a) In the revised version, we added reporting of p-values for the heterogeneity test and two-tailed test p-values for each indicator, as shown in Figs 2-4 as well as Tables 2 and 3. (b) A discussion of the p-values has been added to the "Subgroup moderation effect test" section. The changes are highlighted in red on pages 14-22.

4. In table 1, column Control Group, you mention "regular physical training". What are these? How do these differ with the physical activity in the Experimental Group?

**Response 4**: (a) The column of the control group in Table 1 was incorrectly translated in the original manuscript. We consulted the original studies again in the revised version and checked and translated the extracted information. Table 1 on pages 11-13 has general learn modified areas highlighted in red. (b) On page 10, lines 153-155 of the manuscript, the difference between the control and experimental groups is described. The specifics are as follows: The control group only attended regular physical education classes and did not engage in any other continuous physical activity; the experimental group added only moderate-intensity exercise intervention to the weekly physical education classes.

5. There are many grammatical or potentially conceptual errors in the writing. I am not first English language speaker. But I noticed many grammatical errors.

**Response 5**: We revisited the concept of critical indicators in the revised version and specifically asked native English-speaking experts to enhance the article.

**Respond to the minor points:**

1. Perhaps you want to change the term "literature" to "publication" or "article" or "study".

**Response 1**: In the revised version, we have made some changes to ensure consistency throughout the paper.

2. Sometimes you say "execution" and sometimes you say "executive".

**Response 2**: We asked native English-speaking experts to enhance the text and standardize the usage of relevant words in the revised manuscript; for example, "executive" is used throughout the text.

3. Am I right that all the items in Table 4 include an asterisk? If so, then you don't need to have the asterisk. You can simply mention "P<0.00001" in the caption or notes of the figure

**Response 3**: On pages 16-17, we changed this section to a sensitivity analysis chart to help the reader read the article more clearly.

We appreciate for the reviewer work earnestly on our manuscript, and hope that the correction will meet with approval.

Once again, Thank you very much for your comments and suggestions.

Yours sincerely,

Haixia Wang

9 November 2022
